# Association between profiles of accelerometer-measured daily movement behaviour and mortality risk: a prospective cohort study of British older adults

**DOI:** 10.1136/bmjsem-2023-001873

**Published:** 2024-06-27

**Authors:** Manasa Shanta Yerramalla, Mathilde Chen, Aline Dugravot, Vincent T van Hees, Severine Sabia

**Affiliations:** 1 Université Paris Cité, Inserm U1153, Epidemiology of Ageing and Neurodegenerative Diseases, Paris, France; 2 Division of Clinical Geriatrics, Department of Neurobiology, Care Sciences, and Society, Karolinska Institutet, Stockholm, Sweden; 3 CIRAD, UMR PHIM, Montpellier, France; 4 UMR PHIM, Univ Montpellier, CIRAD, INRAE, Institut Agro, IRD, Montpellier, France; 5 Accelting, Almere, The Netherlands; 6 Faculty of Brain Sciences, UCL, London, UK

**Keywords:** Accelerometer, Aging, Epidemiology, Physical activity, Sedentary

## Abstract

**Objectives:**

We identified profiles of wake-time movement behaviours (sedentary behaviours, light intensity physical activity and moderate-to-vigorous physical activity) based on accelerometer-derived features among older adults and then examined their association with all-cause mortality.

**Methods:**

Data were drawn from a prospective cohort of 3991 Whitehall II accelerometer substudy participants aged 60–83 years in 2012–2013. Daily movement behaviour profiles were identified using k-means cluster analysis based on 13 accelerometer-assessed features characterising total duration, frequency, bout duration, timing and activity intensity distribution of movement behaviour. Cox regression models were used to assess the association between derived profiles and mortality risk.

**Results:**

Over a mean follow-up of 8.1 (SD 1.3) years, a total of 410 deaths were recorded. Five distinct profiles were identified and labelled as ‘active’ (healthiest), ‘active sitters’, ‘light movers’, ‘prolonged sitters’, and ‘most sedentary’ (most deleterious). In model adjusted for sociodemographic, lifestyle, and health-related factors, compared with the ‘active’ profile, ‘active sitters’ (HR 1.57, 95% CI 1.01 to 2.44), ‘light movers’ (HR 1.75, 95% CI 1.17 to 2.63), ‘prolonged sitters’ (HR 1.67, 95% CI 1.11 to 2.51), ‘most sedentary’ (HR 3.25, 95% CI 2.10 to 5.02) profiles were all associated with a higher risk of mortality.

**Conclusion:**

Given the threefold higher mortality risk among those with a ‘most sedentary’ profile, public health interventions may target this group wherein any improvement in physical activity and sedentary behaviour might be beneficial.

**WHAT IS ALREADY KNOWN ON THIS TOPIC**

- Guidelines on wake-time movement behaviours focus primarily on total duration; although movement behaviour is multidimensional in nature and can be further characterised based on dimensions such as intensity, frequency, fragmentation, distribution and timing. Owing to lack of evidence to establish their relevance, especially for older adults, guidelines have given little to no consideration for these other dimensions.

**WHAT THIS STUDY ADDS**

- This study found five movement behaviour profiles that differed according to 13 accelerometer-measured daily movement behaviour features spanning six dimensions.
- The ‘most sedentary’ profile has a threefold higher risk of mortality as compared with older adults with the ‘active’ profile, independent of sociodemographic, lifestyle and health-related risk factors.
- An intermediate similar risk was found for the ‘active sitters’, ‘light movers’ and ‘prolonged sitters’.

**HOW THIS STUDY MIGHT AFFECT RESEARCH, PRACTICE OR POLICY**

- Public health interventions may focus on older adults with the ‘most sedentary’ profile by designing programmes wherein any improvement in physical activity and sedentary behaviour might be beneficial. Around 20% of the study population had an active profile, this shows that this profile can be achieved and be used as the ultimate target.

## Introduction

With increasing life expectancy, older adults represent the fastest growing age group and their proportion with respect to the world’s population is expected to nearly double by 2050.^1^ It is important to understand the drivers of health in this group that is characterised by multimorbity^2 3^ and reduced functioning capacity.^4^ Recent guidelines encourage older adults to engage in at least 150 min per week of moderate-to-vigorous physical activity (MVPA) and reduce time in sedentary behaviour (SB), with no recommendation regarding light intensity physical activity (LIPA).^5^ However, there is low adherence to these guidelines particularly in older adults.^6 7^

The three wake-time movement behaviours—SB, LIPA, MVPA—that make up our days have long been primarily characterised in research settings by their total duration, commonly assessed by questionnaire and their importance for morbidity and mortality^8 9^ is well established, particularly for SB and MVPA. Movement behaviour is multidimensional in nature^10^; in addition to duration, it can be further classified based on dimensions such as intensity, frequency, fragmentation, distribution, and timing.^11 12^ Little to no consideration for these other dimensions is given in the current PA guidelines.^5^ Accelerometers have made it possible to capture these dimensions, and studies have found, for instance, that breaking up prolonged sedentary time^13^ and replacing it with short bouts of PA of any intensity^14^ could improve health. This underlines the importance of investigating different dimensions of movement behaviour.^10^ While these dimensions differ in movement behaviour characterisation, they tend to cluster together within individuals. It is thus important to take into consideration how they combine within a single individual and identify mutually exclusive movement behaviour profiles.

To date, only a few studies^15 16^ have explored the association of movement behaviour profiles with mortality and found that individuals with a combination of high SB, low LIPA, and no or less MVPA are at the most risk. However, these studies had drawbacks such as small sample size^15^ or inclusion of limited movement behaviour characteristics.^16–18^ None of the studies considered the intensity distribution of movement behaviours^19 20^ or the timing of activity,^21 22^ which might be important dimensions for health. Moreover, only one study exclusively focused on older adults, using self-reported measures that cannot capture short, incidental episodes of movement behaviour and examining a single dimension, duration in movement behaviours.^16^ To the best of our knowledge, no study has investigated the association of movement behaviour profiles comprising different dimensions of objectively measured movement behaviours with mortality risk in older adults. Therefore, this study aimed to identify mutually exclusive profiles of daily movement behaviour based on 13 objectively measured PA and SB characteristics and to examine their association with all-cause mortality among older adults. Based on the literature we expect a set of profiles ranging from the most sedentary, characterised by worst values on all SB dimensions to the most active, composed of the most favourable values on each MVPA dimension; although we have no hypothesis regarding how features related to duration, frequency, and fragmentation of LIPA, as well as timing and activity distribution, will contribute to the movement behaviour profiles; we hypothesise the most sedentary profile to be associated with the highest risk of all-cause mortality among older adults.

## Materials and methods

### Study design and participants

The Whitehall II study is an ongoing prospective cohort established in 1985–1988 among 10 308 London-based civil servants (67% males) aged 35–55 years.^23^ Since study inception, sociodemographic, lifestyle, and health-related factors have been assessed using questionnaires and clinical examinations. Subsequent follow-up assessments have taken place at approximately every 4–5 years since baseline. The accelerometer substudy was undertaken during the 2012–2013 wave of data collection for the 4880 participants seen at the London clinic or living in the South-Eastern regions of England who underwent clinical examinations at home.

### Patient and public involvement

There has been no patient and public involvement in research methods.

### Accelerometer measurement

Participants without any contraindications (ie, allergies to metal or plastic, travelling abroad in the subsequent week) were asked to wear a research-grade triaxial accelerometer (GENEActiv Original; Activinsights, Kimbolton, UK; https://activinsights.com/wp-content/uploads/2022/06/GENEActiv-Product-Information-Sheet.pdf) on their non-dominant wrist during nine consecutive days over 24 hours. Data were sampled at 85.7 Hz, expressed relative to gravity (1 *g*=9.81 m/s^2^) and processed using GGIR R package^24^ V.2.4-1 (https://CRAN.R-project.org/package=GGIR). Data were corrected for calibration error^25^ and Euclidean norm of raw accelerations minus one with negative numbers rounded to 0 was calculated.^26^ Sleep periods were detected using a validated algorithm, which was guided by sleep log.^27^ Data from day 2 to day 8 were retained, corresponding to seven full days. For each day, the waking period, defined as the period between waking and onset of sleep, was retained for the analysis. Participants were included if accelerometer wear time was ≥2/3 of the waking period for at least 2 weekdays and 2 weekend days.^28^ Non-wear period among valid days (accelerometer wear time ≥2/3 of the waking period) was corrected based on a previously reported algorithm.^26^

### Characteristics of accelerometer-assessed daily movement behaviour

Based on existing literature accounting both for evidence of associations with health and most commonly used features, we chose a set of 13 accelerometer-derived movement behaviour features allowing to capture the following 6 movement behaviour dimensions: overall activity level, total duration and^29^ frequency, typical duration (also marker of fragmentation),^30^ activity intensity distribution^19^ and timing of PA.^21 22^ Table 1 describes which features represent each of the dimension. For each participant, all features were derived over each waking period and averaged over 7 days. For those with <7 valid days, a weighted average was computed using information on the number of week and weekend days.^28^

**Table 1:** Description of features of daily movement behaviours

Dimension	Feature	Description
Overall activity level	Average acceleration in m*g*	Average acceleration during the waking period, which is a global metric for overall activity level.
Total duration	Total duration of SB Total duration of LIPA Total duration of MVPA in minutes per day	Total durations of SB, LIPA and MVPA corresponded to the total time during waking period with average acceleration over 60 s epoch length*<40 m*g*, 40–99 m*g* and ≥100 m*g*, respectively.
Frequency	Number of sedentary bouts Number of LIPA bouts Number of MVPA bouts	Number of bouts† (episodes) of uninterrupted time spent in the specific movement behaviour. This is a measure of fragmentation of total daily duration of movement behaviour.
Typical duration	Mean duration of sedentary bouts Mean duration of LIPA bouts Mean duration of MVPA bouts in minutes	Average length of bout duration for each of the movement behaviour computed as the total daily duration divided by daily number of bouts. It is a marker of activity fragmentation, that is the propensity to transition from one movement behaviour (SB, LIPA, or MVPA) to another.^46^ For example, lower the mean duration of SB bouts, more fragmented accumulation pattern of sedentary time.
Activity intensity distribution	Intensity constant Intensity gradient	Based on the linear relationship between log of intensity and log of time in that intensity across the waking period. For example, when the constant is lower and the gradient is less negative, time accumulated during waking period is more evenly distributed across the intensity spectrum.
Timing	Timing of the most active 5 hours in hours	Denotes the timing of the start of the 5 hours period with the maximum average acceleration. These periods are estimated using a rolling 5 hours window. For example, a value of 7 represents that the most active 5 consecutive hours start at 7:00 hours until noon.

*The epoch length refers to the duration over which mean acceleration is aggregated.^47^

†A bout refers to a continuous episode of a movement behaviour without interruptions. It can last between 60 s (epoch length) and the maximum duration spent in an episode of a given movement behaviour.

LIPA, light intensity physical activity; m*g*, milligravity; MVPA, moderate-to-vigorous physical activity; SB, sedentary behaviour.

### Mortality ascertainment

All-cause mortality was assessed until 28 February 2021, through the UK national mortality register kept by the National Health Service (NHS) Central Registry. Tracing exercise was conducted by using each participants’ unique NHS identification number. Participants were followed from the date of clinical examination at 2012–2013 wave until the record of death or the end of follow-up, whichever came first.

### Covariates

Covariates were assessed by questionnaire or at clinical examination during 2012–2013 wave of data collection, as well as from electronic health records including Hospital Episode Statistics and the Mental Health Services dataset. Sociodemographic variables included sex, ethnicity, marital status, education, and last known occupational position. Lifestyle factors consisted of smoking status, alcohol consumption, and fruit and vegetable consumption. Health-related factors comprised cardiometabolic risk factors (body mass index (BMI), hypertension, hyperlipidaemia, and diabetes) and a morbidity index calculated as the count of the following chronic conditions: coronary heart disease, stroke, heart failure, cancer, arthritis, chronic obstructive pulmonary disease, depression, Parkinson disease, and dementia. Details on covariates are provided in online supplemental eMethods.

10.1136/bmjsem-2023-001873.supp1 Supplementary data

### Statistical analysis

Profiles of accelerometer-assessed features were identified using the k-means clustering algorithm.^31 32^ As compared with other clustering techniques, k-means allows for partitioning participants into *k* non-nested and exclusive groups.^32^ Given its low complexity, ability in handling large dataset, and fast calculation, k-means is one of the most widely used technique in clustering.^33^ Clustering was performed so that participants within a specific group are as similar as possible (high intraclass similarity) and participants within one group are as dissimilar as possible to the participants in other groups (low interclass similarity).^32^ All 13 features were standardised (mean=0, SD=1) and included in the k-means algorithm.^31^ To choose the appropriate number of profiles, a range of possible solutions was first determined using the elbow method^34^ and gap statistic method (online supplemental eMethods)^35^ as selection criteria. The degree of similarity within a group (within-cluster sum of squares) and dissimilarity between groups (between-cluster sum of squares) were assessed for the different identified profile sizes. In order to describe and name each profile, we examined the mean standardised movement behaviour features within each group. Each variable was interpreted in relative terms by comparing to the mean of the said feature; this follows the established trend by prior research.^15^

A one-way analysis of variance (ANOVA) test was conducted to assess the mean differences in accelerometer-assessed features across the identified profiles. If the differences between profiles were significant, a Tukey post hoc test was performed to determine pairwise mean differences between each profile.

Cox proportional hazard model was then used to estimate the HRs and 95% CIs for the association of each of the 13 movement behaviour features (in exploratory analysis) and of profiles of movement behaviour (main analysis) with risk of all-cause mortality. The proportionality assumption was assessed using Schoenfeld’s test. Analyses were first adjusted for age (used as timescale) and sociodemographic risk factors, then further adjusted for lifestyle factors and finally for health-related factors. Interaction between each of the profiles with age (continuous), sex, obesity (BMI<30 kg/m^2^ and ≥30 kg/m^2^) and morbidity (0 and ≥1 disease) were also tested. The main analysis was conducted with the profile with the highest overall activity level as the reference. In order to compare risks between all profiles, fully adjusted models were then repeated using each profile as the reference group. In a sensitivity analysis, deaths occurring within the first 2 years of follow-up were excluded to examine potential reverse causation. All analyses were undertaken using STATA statistical software V.15 (StataCorp) and R V.3.6.1 (https://www.r-project.org) for cluster analysis. For all tests reported in the results section, a two-sided p<0.05 was considered as statistically significant.

## Results

Of the 6308 participants in the 2012–2013 wave, 4880 were invited to participate in the accelerometer substudy, 4492 agreed, and 4008 returned the devices with valid data (accelerometer wear time ≥2/3 of the waking period for at least 2 weekdays and 2 weekend days). Excluding those with missing covariates (N=17) led to an analytical sample of 3991 participants (online supplemental eFigure 1). Compared with participants included in the analysis (N=3991), those excluded (N=889) were more likely to be younger and have higher education (online supplemental eTable 1). Among the 3991 study participants, 410 deaths were recorded over a mean follow-up of 8.1 (SD=1.3) years.

In exploratory analysis examining the association between each of the 13 movement behaviour features with mortality risk, we found all features, except timing of the most active 5 hours, to be associated with mortality risk in models adjusted for sociodemographic factors. These associations remained in models further adjusted for lifestyle and health-related factors (all p<0.02), except that of the mean duration of LIPA (online supplemental eTable 2). The moderate to high correlation between each feature (online supplemental eTable 3), apart from ‘timing of the most active 5 hours’ and ‘number of MVPA bouts’, motivated us to move forward to derive profiles of movement behaviour to account for the dependency between the features.

The optimal number of profiles ranged from 3 to 5 according to the selection criteria (online supplemental eFigure 2). The 5-profile solution had the minimum within-cluster sum of squares and the maximum between-cluster sum of squares (online supplemental eTable 4) and provided meaningfully distinct profiles and was selected as the optimal number of profiles.

**Figure 1:**
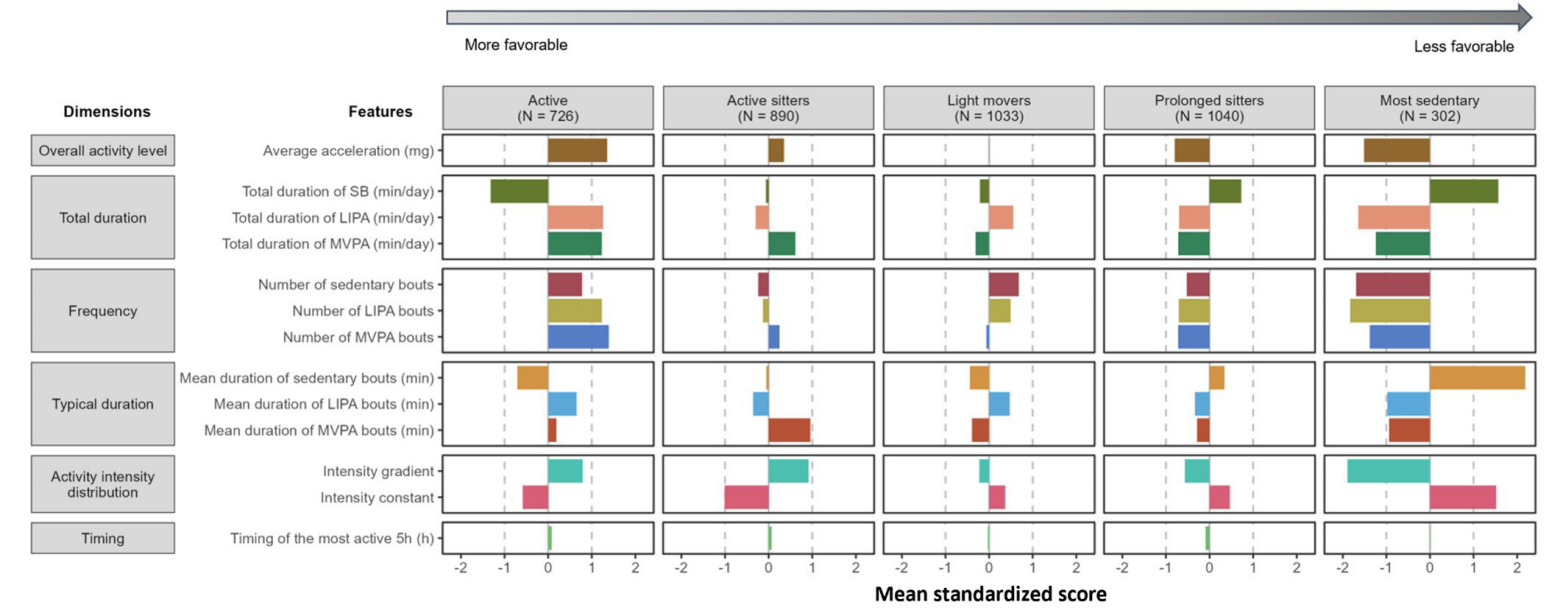
Mean standardised values of 13 features of daily movement behaviour by profile. Mean value of 0 corresponds to the average observed value in the study population (mean and SD in the study population are displayed in table 2). Positive values represent higher acceleration, higher total duration in SB, LIPA and MVPA, higher number of bouts, higher mean duration of bouts, higher intensity gradient, higher intensity constant, and later timing of activity. LIPA, light intensity physical activity; MVPA, moderate-to-vigorous physical activity; SB, sedentary behaviour.

Figure 1 presents the standardised values for the 13 features for each of the 5 profiles (numbers displayed in online supplemental eTable 5). The retained profiles were named: active, active sitters, light movers, prolonged sitters, and most sedentary. Table 2 presents the mean of the 13 features for each of the 5 profiles. ‘Active’ (N=726 (18.2% of the total sample)) profile was characterised by the lowest total duration and most fragmented SB (as denoted by shorter and more frequent sedentary bouts), coupled with the highest overall activity and total duration of LIPA and MVPA, compared with other profiles. ‘Active sitters’ (890 (22.3%)) had longer MVPA bouts and a more uniformly distributed time across the intensity spectrum, compared with other profiles but also had higher SB duration and less frequently interrupted SB than ‘active’ profile. ‘Light movers’ (1033 (25.9%)) spent more time in LIPA and less in SB but had worse MVPA attributes as compared with participants from the ‘active’ profile. ‘Prolonged sitters’ (1040 (26.1%)) had the second-worst scores for all characteristics defining SB and had more interruptions of SB than the worst profile, ‘most sedentary’ (302 (7.6%)), showing 10 out of the 13 movement behaviour features being 1-SD from the mean. All 13 movement behaviour features differed between profiles (p<0.001 for all variables in ANOVA tests). Online supplemental eFigure 3 shows the distribution of participants in the 5 profiles on the 2 first principal components from a principal component analysis of the 13 features (the description of the 2 principal components, explaining 74.7% of the variance, is provided in online supplemental eFigure 4). This shows the increase in overall activity level from the ‘most sedentary’ to the ‘active’ profiles, with profiles ‘light movers’ and ‘active sitters’ differing also on the way they are active (LIPA vs MVPA).

**Table 2:** Characterisation of the five identified profiles of daily movement behaviours

Daily movement behaviour features	Overall (N=3991)	Daily movement behaviour profiles				
		Active (N=726)	Active sitters (N=890)	Light movers (N=1033)	Prolonged sitters (N=1040)	Most sedentary (N=302)
Overall activity level						
Average acceleration (m*g*)	31.8 (9.7)	44.9 (7.6)*	35.2 (7.6)*	31.7 (3.1)*	24.1 (2.6)*	17.2 (2.8)*
Total duration (min/day)						
Total duration of SB (min/day)	717.9 (100.1)	585.8 (60.7)*	711.8 (62.0)*	696.7 (54.1)*	790.7 (56.4)*	875.2 (69.2)*
Total duration of LIPA (min/day)	210.3 (69.1)	297.2 (49.3)*	189.6 (33.2)*	248.8 (35.4)*	162.1 (30.8)*	96.6 (33.0)*
Total duration of MVPA (min/day)	56.1 (38.6)	103.6 (35.8)*	79.7 (29.1)*	44.2 (16.5)*	28.4 (13.1)*	8.2 (6.6)*
Frequency						
Number of sedentary bouts	71.8 (16.0)	84.3 (12.2)*	68.0 (9.6)*	82.8 (11.3)*	63.4 (10.2)*	44.7 (12.9)*
Number of LIPA bouts	86.0 (21.6)	112.6 (12.6)^*^	83.1 (11.0)*	96.7 (10.9)*	70.7 (10.4)*	46.4 (13.4)*
Number of MVPA bouts	23.2 (13.6)	42.1 (11.6)*	26.6 (9.0)*	22.4 (7.3)^*^	13.5 (5.5)*	4.5 (3.1)*
Typical duration						
Mean duration of sedentary bouts (min)	11.5 (5.9)	7.3 (1.3)*	11.2 (2.2)*	8.9 (1.4)*	13.5 (2.8)*	24.4 (12.7)*
Mean duration of LIPA bouts (min)	2.4 (0.4)	2.6 (0.4)*	2.3 (0.3)† ‡ §	2.6 (0.3)*	2.3 (0.3)† ‡ §	2.0 (0.3)*
Mean duration of MVPA bouts (min)	2.3 (0.9)	2.5 (0.6)*	3.2 (1.1)*	1.9 (0.4)*	2.0 (0.6)*	1.4 (0.9)*
Activity intensity distribution						
Intensity constant	12.4 (0.7)	12.0 (0.4)*	11.7 (0.5)*	12.6 (0.3)*	12.7 (0.4)*	13.4 (0.5)^*^
Intensity gradient	−2.10 (0.20)	−1.91 (0.12)*	−1.88 (0.13)*	−2.13 (0.10)*	−2.21 (0.12)*	−2.49 (0.18)*
Timing						
Timing of the most-active 5 hours	10.2 (1.6)	10.4 (1.7)¶	10.3 (1.7)¶	10.2 (1.5)	10.1 (1.5)† **	10.2 (1.9)

Values are mean (SD).

*Indicates significant difference from all other profiles in pairwise comparison.

†Indicates significant difference from active.

‡Indicates significant difference from light movers.

§Indicates significant difference from most sedentary.

¶Indicates significant difference from prolonged sitters.

**Indicates significant difference from active sitters.

LIPA, light intensity physical activity; MVPA, moderate-to-vigorous physical activity; SB, sedentary behaviour.

Table 3 shows the sociodemographic, lifestyle and health-related characteristics of each movement behaviour profile. Participants in the ‘most sedentary’ profile were the oldest, had the highest proportion of non-white participants, were more likely to be single, from lower occupational position, smokers, and had the worst cardiometabolic and morbidity profile, as compared with other profiles (p<0.001). Compared with ‘prolonged sitters’, ‘light movers’ tended to be younger, women, had better diet, and a better cardiometabolic profile (p<0.001). ‘Most sedentary’ profile had the highest proportion of deaths (30.1%), followed by ‘prolonged sitters’ (12.1%), ‘light movers’ (10.2%), ‘active sitters’ (6.4%) and ‘active’ (4.3%) (p<0.001).

**Table 3:** Baseline characteristics by profiles of daily movement behaviours

	Overall (N=3991)	Daily movement behaviour profiles				
		Active (N=726)	Active sitters (N=890)	Light movers (N=1033)	Prolonged sitters (N=1040)	Most sedentary (N=302)
Age (years), M (SD)	69.4 (5.7)	67.5 (4.8)* † ‡	67.2 (4.8)* † ‡	69.8 (5.6)§	71.0 (5.8)§	73.6 (5.6)§
Women	1030 (25.8)	214 (29.5)¶ †	135 (15.2)§	336 (32.5)¶ †	250 (24.0)§	95 (31.5)¶ †
Non-white	295 (7.4)	42 (5.8)† ‡	39 (4.4)* † ‡	73 (7.1)¶ ‡	95 (9.1)§	46 (15.2)§
Married/cohabitating	2981 (74.7)	561 (77.3)‡	682 (76.6)‡	792 (76.7)¶ ‡	764 (73.5)¶ * ‡	182 (60.3)§
Higher education	1238 (31.0)	219 (30.2)¶	344 (38.7)§	280 (27.1)¶	318 (30.6)¶	77 (25.5)¶
Low occupational position	2013 (50.9)	370 (51.0)¶ ‡	377 (42.4)§	562 (54.4)¶ ‡	538 (51.7)¶ ‡	184 (60.9)§
Recent-ex/current smokers	221 (5.5)	29 (4.0)† ‡	41 (4.6)† ‡	50 (4.8)¶ ‡	67 (6.4)** ¶ ‡	34 (11.3)§
>14 units of alcohol per week	925 (23.2)	198 (27.3)* † ‡	232 (26.1)† ‡	233 (22.6)** ¶ ‡	221 (21.3)** ¶ ‡	41 (13.6)§
Daily fruit and vegetable intake	3165 (79.3)	585 (80.6)†	743 (83.5)* † ‡	824 (79.8)¶ †	786 (75.6)** ¶ ‡	227 (75.2)¶
Body mass index ≥30 kg/m^2^	723 (18.1)	63 (8.7)§	108 (12.1)§	191 (18.5)§	260 (25.0)§	101 (33.4)§
Hypertension	2066 (51.8)	287 (39.5)* † ‡	387 (43.5)* † ‡	525 (50.8)§	649 (62.4)§	218 (72.2)§
Hyperlipidaemia	2021 (50.6)	308 (42.4)* † ‡	403 (45.3)* † ‡	540 (52.3)** ¶ ‡	588 (56.5)** ¶	182 (60.3)** ¶
Diabetes	514 (12.9)	49 (6.8)* † ‡	68 (7.6)* † ‡	130 (12.6)§	195 (18.8)** ¶ ‡	72 (23.8)** ¶ ‡
Morbidity index, M (SD)††	0.5 (0.8)	0.4 (0.6)§	0.4 (0.6)§	0.6 (0.8)** ¶ ‡	0.6 (0.8)** ¶ ‡	0.9 (0.9)§
Number of deaths	410 (10.3)	31 (4.3)* † ‡	57 (6.4)* † ‡	105 (10.2)** ¶ ‡	126 (12.1)** ¶ ‡	91 (30.1)§

Values are N (column %), unless otherwise stated.

*Indicates significant difference from light movers.

†Indicates significant difference from prolonged sitters.

‡Indicates significant difference from most sedentary.

§Indicates significant difference from all other profiles in pairwise comparison.

¶Indicates significant difference from active sitters.

**Indicates significant difference from active.

††Number of chronic conditions among coronary heart disease, stroke, heart failure, cancer, arthritis, chronic obstructive pulmonary disease, depression, Parkinson disease and dementia.

M, mean.

There was no evidence of effect modification by age, sex, obesity, or morbidity status (p≥0.13 for all interactions) so analyses were conducted in the full study sample. The proportional hazards assumption was not violated (p=0.42). Table 4 shows the association between the 5 profiles of movement behaviour and all-cause mortality sequentially adjusted for sociodemographic, lifestyle and health-related risk factors. In the fully adjusted model, compared with the ‘active’ profile, other profiles were associated with a higher risk of all-cause mortality risk with increasing risk from the most to less active profiles. HRs (95% CIs) were 1.57 (1.01 to 2.44; p=0.04) for ‘active sitters’, 1.75 (1.17 to 2.63; p=0.01) for ‘light movers’, 1.67 (1.11 to 2.51; p=0.01) for ‘prolonged sitters’ and 3.25 (2.10 to 5.02; p<0.001) for the ‘most sedentary’ profile.

**Table 4:** Association between profiles of daily movement behaviours and all-cause mortality (N total=3991, N cases=410, mean follow-up (SD)=8.1 (1.3) years)

Profiles	N cases/N total	HR (95% CI)		
		Model adjusted for sociodemographic factors*	Additionally adjusted for lifestyle factors†	Additionally adjusted for health-related factors‡
Active	31/726	1.00 (Reference)	1.00 (Reference)	1.00 (Reference)
Active sitters	57/890	1.54 (1.00 to 2.40)	1.55 (1.00 to 2.41)	1.57 (1.01 to 2.44)
Light movers	105/1033	1.76 (1.18 to 2.64)	1.76 (1.18 to 2.63)	1.75 (1.17 to 2.63)
Prolonged sitters	126/1040	1.76 (1.18 to 2.62)	1.69 (1.14 to 2.53)	1.67 (1.11 to 2.51)
Most sedentary	91/302	3.58 (2.35 to 5.45)	3.43 (2.25 to 5.24)	3.25 (2.10 to 5.02)

*Models adjusted for age (time-scale), sex, ethnicity, marital status, education and last occupational position.

†Models additionally adjusted for smoking status, alcohol consumption, and fruit and vegetable consumption.

‡Models additionally adjusted for body mass index, hypertension, hyperlipidaemia, diabetes and morbidity index.

In fully adjusted models with different reference categories (online supplemental eTable 6), the risk of mortality was the greatest among the ‘most sedentary’ profile while no differences were seen between ‘active sitters’, ‘light movers’ and ‘prolonged sitters’. In the sensitivity analysis, excluding deaths within the first 2 years (N=45) showed broadly similar results (online supplemental eTable 7), although the association with ‘active sitters’ was no longer significant in the fully adjusted model (1.47 (95% CI: 0.93 to 2.33; p=0.10)).

## Discussion

In this longitudinal study of 3991 British older adults followed for more than 8 years, 5 distinct movement behaviour profiles were identified from 13 accelerometer-derived variables characterising overall activity level, total duration, frequency, typical duration, activity intensity distribution, and timing of wake-time movement behaviour using k-means cluster analysis. The identified profiles were ‘active’, ‘active sitters’, ‘light movers’, ‘prolonged sitters’, and ‘most sedentary’. The ‘active’ profile had the lowest duration and most fragmented accumulation pattern of sedentary time, and the highest duration of PA compared with the other four profiles. Independent of sociodemographic, lifestyle, and health-related factors, we observed a non-linear increase of all-cause mortality risk across the profile levels moving from the healthiest, ‘active’, to the worst, ‘most sedentary’, profiles. Compared with participants in the ‘active’ profile, ‘light movers’ and ‘prolonged sitters’ had a similar 70% higher mortality risk while a threefold higher risk was found among ‘most sedentary’.

Up until recently, most studies examined profiles based on a priori categorisation of 1 or 2 dimensions of movement behaviours such as duration and/or fragmentation.^17 36^ A recent study on 2021 older adults identified PA phenotypes using distributional representations of time in accelerometer-derived activity intensity; however, the clinical phenotypes were based on the single dimension of total duration of movement behaviours without consideration for any other dimension.^18^ To the best of our knowledge, one previous study in a sample population of 851 participants (mean age=53 years) followed up over 15 years aimed to identify mutually exclusive profiles of 14 accelerometer-derived variables—covering dimensions of total and variation of time in SB, LIPA, and MVPA across days of the week, time in SB and MVPA bouts, and overall activity level— and investigated their association with mortality.^15^ That study derived 3 profiles and found that compared with the ‘low active’ profile, the ‘average’ and ‘high active’ profiles were associated with lower mortality risk.^15^ Contrarily to the present findings, they found no difference in mortality risk between the latter 2 profiles.^15^ Another approach found in the literature focuses on a priori combinations of time in MVPA and time in SB. A meta-analysis of such accelerometer-based studies examined their association with mortality risk and reported that the highest risk was among those with lowest time in MVPA and highest time in SB, with intermediate risk in those with lowest time in MVPA and low/intermediate time in SB, or intermediate time in MVPA and highest time in SB.^17^ In the present study, using 13 accelerometer-derived features covering 6 dimensions of movement behaviour, we identified 5 profiles of movement behaviour among older adults, the ‘active’ profile showing the lowest risk of mortality, the ‘most sedentary’ the highest and the three others intermediate risk.

In our study, the profiles of ‘light movers’ and ‘prolonged sitters’ have similar risk for mortality. The largest differences between these 2 groups were for SB and LIPA characteristics. ‘Light movers’ had lower SB than ‘prolonged sitters’ and the second-best LIPA parameters. Interventions among older adults are conducted wherein engagement in LIPA has been proposed as an alternative for MVPA, especially for the most sedentary group given that LIPA might be easier to initiate for this group.^37^ Recent studies also suggest that replacing SB with LIPA might be beneficial in reducing mortality risk for older adults.^38^ This has been reflected in the recent WHO 2020 guidelines on PA and SB which recommend replacing sedentary time with PA of any intensity, including LIPA.^39^ However, findings from the present study suggest that a balanced approach with regard to LIPA and MVPA might be seen as a final target.

When comparing to the ‘most sedentary’ profile, we found that ‘prolonged sitters’ profile was associated with 51% lower mortality risk. The major differences between these profiles were the high absolute duration of SB among the ‘most sedentary’ profile and the least fragmented SB throughout the day. This is somewhat in line with an earlier study examining the day-to-day variations in characteristics of SB duration and bouts, separately, which observed a higher mortality risk in the category with the highest percentage of prolonged (>30 min) sedentary bouts as compared with other 6 categories with gradually lower percentage of prolonged sedentary bouts over days of the week.^40^ Interestingly, the main differences between the 2 most active profiles, ‘active’ and ‘active sitters’, were also the total time in SB and the way SB and PA accumulate (both in terms of frequency and mean duration of bouts). We found that participants with ‘active’ profile had lower mortality risk than ‘active sitters’, highlighting the importance of these characteristics also in the most active population.

### Strengths and limitations

Our study has several strengths. Movement behaviour features were assessed objectively, and the profiles were derived using a robust analytical method. We also considered an extensive range of dimensions, unlike previous studies. This study controlled for a wide range of risk factors, such as diabetes and multimorbidity, which were ascertained using various objective sources such as clinical examinations and record linkage data.

This study has also limitations. Wrist-worn accelerometers might not capture adequately some types of activities, such as cycling or carrying groceries while walking.^41^ They do not provide information on posture and cannot distinguish between sitting and ‘passive’ standing positions which could lead to misclassification between SB and inactivity.^42^ However, wrist accelerometers have been found to classify movement behaviours based on metabolic intensity with accuracy.^43^ The Whitehall II study was originally an occupational cohort wherein the participants were healthier than the general population, however, it has previously been shown that the associations of various cardiovascular risk factors, including PA, with incident cardiovascular disease from this cohort were similar to that of other general population cohorts.^44^ The ethnic distribution in the study reflects the UK population 30 years ago, and the study lacks sufficient numbers to allow analyses for specific minority groups. Replication studies across cultures are required to account for differences in healthcare systems and lifestyle preferences to better assess the generalisability of our findings. Finally, data on socioeconomic and lifestyle covariates such as dietary intake and smoking status were self-reported and recall and social desirability biases might not be excluded.^45^

## Conclusions

The present study highlights that not just the total duration in activity levels, but also other characteristics—such as the manner in which SB and PA accumulate throughout the day and are distributed—are essential parts of an individual’s movement behaviour profile. Among older adults, we identified the ‘most sedentary’ profile as having the greatest mortality risk. This group constitutes a high-risk category, warranting interventions that specifically address their needs. In contrast, the ‘active’ group had the lowest risk, it was characterised by <10 hours of SB, around 5 hours of LIPA and >1 hour 30 min of MVPA per day, but also by more fragmented SB with the mean duration of SB bout lasting <10 min, and a well-balanced distribution of activity over the intensity spectrum. Considering that approximately one-fifth of older adults in the present study exhibited this ‘active’ profile, demonstrating achievability, public health initiatives should emulate this as the ultimate target.

10.1136/bmjsem-2023-001873.supp2 Supplementary data

## Data Availability

Data, protocols and other metadata of the Whitehall II study are available to the scientific community either via the Whitehall II study data sharing portal (https://www.ucl.ac.uk/epidemiology-health-care/research/epidemiology-and-public-health/research/whitehall-ii/data-sharing). All scripts to conduct k-means clustering and produce the figures are openly accessible in the following repository: https://github.com/MathildeChen/PCA-K-means-for-PA-features.
