# Supplementary material for: Association between profiles of accelerometer-measured daily movement behaviour and mortality risk: a prospective cohort study of British older adults
Source: BMJ Open Sport Exerc Med. 2024 Jun 27;10(2):e001873. doi: 10.1136/bmjsem-2023-001873 (PMC11216065; doi:10.1136/bmjsem-2023-001873)
Supplement: Supplementary data [file bmjsem-2023-001873supp001.pdf]

SUPPLEMENT

Association between profiles of accelerometer-measured daily movement behaviour and mortality risk: a prospective cohort study of British older adults

Manasa Shanta Yerramalla,[1,2]* Mathilde Chen,[1,3,4]* Aline Dugravot,[1] Vincent T van Hees,[5] Séverine Sabia,[1,6]

[1]Université Paris Cité, Inserm U1153, Epidemiology of Ageing and Neurodegenerative diseases, Paris, France
[2]Division of Clinical Geriatrics, Department of Neurobiology, Care Sciences, and Society, Karolinska Institutet, Stockholm, Sweden
[3]CIRAD, UMR PHIM, F-34398 Montpellier, France
[4]PHIM, Univ Montpellier, CIRAD, INRAE, Institut Agro, IRD, Montpellier, France
[5]Accelting, Almere, The Netherlands
[6]Faculty of Brain Sciences, University College London, UK

[†]Manasa Shanta Yerramalla, and Mathilde Chen are equally contributing first authors.

TABLE OF CONTENTS

eMETHODS 2
Covariates 2
Determination of the range of possible number of profiles and software/package used for the analysis 3

SUPPLEMENTARY TABLES 4
eTable 1 Characteristics of participants included and excluded from the analysis 4
eTable 2 Association between each feature of daily movement behaviour and all-cause mortality (N total = 3991, N cases = 410, mean follow-up (standard deviation) = 8.1 (1.3) years) 5
eTable 3 Correlation matrix of 13 features of daily movement behaviours 6
eTable 4 Parameters assessing the variability of participants within and between clusters for different profiles 7
eTable 5 Standardized values of movement behaviour variables by profiles 8
eTable 6 Association between profiles of movement behaviours and all-cause mortality, with different reference categories (N total = 3991, N cases = 410, mean follow-up (SD) = 8.1 (1.3) years) 9
eTable 7 Association between profiles of movement behaviours and all-cause mortality using 2-year washout period (N total = 3946, N cases = 365, mean follow-up (SD) = 6.2 (1.04) years) 10

SUPPLEMENTARY FIGURES 111
eFigure 1 Participant flow chart 11
eFigure 2 Determination of the optimal number of clusters 12
eFigure 3 Visualization of the five profiles of daily movement behaviours using the initial two principal components of the principal component analysis 13
eFigure 4 Loadings of movement behaviour features in the two first principal components used to describe the identified movement behaviour clusters in eFigure 3 14

Yerramalla MS, *et al. BMJ Open Sp Ex Med* 2024; 10:e001873. doi: 10.1136/bmjsem-2023-001873

eMETHODS

Covariates

Covariates were assessed by questionnaire or at clinical examination during 2012-2013 wave of data collection, as well as from electronic health records including HES and the Mental Health Services dataset. Sociodemographic variables consisted of sex, ethnicity (white, non-white), marital status (married/cohabitating, divorced/widowed/single), education (≤primary school, lower secondary, higher secondary school, university, higher degree; treated as a continuous variable), and last known occupational position (high, intermediate, low). Lifestyle factors consisted of fruit and vegetable consumption (less than once daily, once daily, more than once daily), smoking status (current and recent ex-(less than 5 years) smokers, long term ex-smokers, never smokers), and alcohol consumption (0, 1-14, >14 units per week). Health-related factors comprised cardiometabolic factors and a morbidity index. Cardiometabolic factors included body mass index (BMI; categorized as <24.9, 25-29.9 and ≥30 kg/m2), prevalent diabetes (fasting glucose ≥7.0 mmol/l or self-reported doctor diagnosis or use of diabetes medication or hospitalizations ascertained through record linkage to HES (ICD-9 codes 250 or ICD-10 code E11), hypertension (systolic/diastolic blood pressure ≥140/90 mmHg or use of antihypertensive drugs), and hyperlipidaemia (low-density lipoproteins (LDL) >4.1 mmol/l or use of lipid-lowering drugs). A morbidity index was calculated as the number of the following chronic conditions: coronary heart disease, stroke, heart failure, cancer, arthritis, chronic obstructive pulmonary disease, depression, Parkinson disease, and dementia.

Yerramalla MS, *et al. BMJ Open Sp Ex Med* 2024; 10:e001873. doi: 10.1136/bmjsem-2023-001873

Determination of the range of possible number of profiles and software/package used for the analysis

The user must specify the number of profiles (k) in the k-means clustering approach. It is recommended to combine the findings of many procedures rather than relying on a single rule to determine the number of profiles (clusters).[1] As a result, two different indices were utilized to determine the appropriate range of number of clusters to be examined.

A) The Elbow method selects the number of clusters to be such that adding an additional cluster does not significantly reduce the within-group sum of squares, which quantifies the degree to which items within a cluster are similar, representing a trade-off between a reasonable number of clusters and clustering quality.

B) Gap statistic compares the clusters created from the observed data and clusters created from a randomly generated dataset, known as the reference dataset. For a given *k*, the gap statistic is the difference in the total within-cluster variance for the observed data and that of the reference dataset. The optimal number of clusters is denoted by the value of *k* that yields top the largest gap statistic.[2]

Cluster analysis was undertaken in R (version 3.6.1, http://www.r-project.org/) using the *kmeans()* function with 25 different random starting assignments (*nstart = 25*). The Elbow method, and gap statistic tests were performed using the *fviz_nbclust()* function of the factoextra package of R (version 1.0.7, https://CRAN.R-project.org/package=factoextra). Tukey tests for multiple means comparison were performed using the *cld()* function from the multicomp package (version 1.4-16, https://cran.r-project.org/web/packages/multcomp/multcomp.pdf).

References

1. Everitt BS, Landau S, Leese M, et al. Cluster analysis 5th ed: John Wiley, 2011.
2. Tibshirani R, Walther G, Hastie T. Estimating the number of clusters in a data set via the gap statistic. *Journal of the Royal Statistical Society: Series B (Statistical Methodology)* 2001;63(2):411-23.

SUPPLEMENTARY TABLES

eTable 1 Characteristics of participants included and excluded from the analysis

Characteristics	Included in the study sample		
	No (N=889)	Yes (N=3991)	P value
N (row %)	889 (18.2)	3991 (81.8)	
Age (years), M (SD)	68.9 (5.6)	69.4 (5.7)	0.03
Women	298 (33.5)	1030 (25.8)	<0.001
Non-white	93 (10.5)	395 (7.4)	<0.01
Married/cohabitating	654 (73.6)	2981 (74.7)	0.49
University or higher degree	325 (36.6)	1238 (31.0)	<0.01
Low occupational position	443 (49.8)	2031 (50.9)	0.57

Values are N (column %), unless otherwise stated.

Abbreviations: M, mean; SD, standard deviation.

Yerramalla MS, *et al. BMJ Open Sp Ex Med* 2024; 10:e001873. doi: 10.1136/bmjsem-2023-001873

eTable 2 Association between each feature of daily movement behaviour and all-cause mortality (N total = 3991, N cases = 410, mean follow-up (standard deviation) = 8.1 (1.3) years)

	**Hazard ratio (95% confidence interval)**		
**Daily movement behaviour features**	**Model adjusted for sociodemographic factors***	**Additionally adjusted for lifestyle factors†**	**Additionally adjusted for health-related factors‡**
Average acceleration (m*g*)	0.967 (0.955 to 0.979)	0.969 (0.957 to 0.982)	0.972 (0.959 to 0.985)
Total duration of SB (min/day)	1.002 (1.001 to 1.003)	1.002 (1.001 to 1.003)	1.002 (1.001 to 1.003)
Total duration of LIPA (min/day)	0.997 (0.995 to 0.998)	0.997 (0.996 to 0.999)	0.997 (0.996 to 0.999)
Total duration of MVPA (min/day)	0.991 (0.987 to 0.994)	0.991 (0.988 to 0.995)	0.992 (0.989 to 0.996)
Number of sedentary bouts	0.988 (0.982 to 0.993)	0.989 (0.983 to 0.995)	0.99 (0.985 to 0.996)
Number of LIPA bouts	0.989 (0.984 to 0.993)	0.99 (0.985 to 0.994)	0.991 (0.986 to 0.995)
Number of MVPA bouts	0.976 (0.967 to 0.985)	0.978 (0.969 to 0.987)	0.98 (0.971 to 0.990)
Mean duration of sedentary bouts (min)	1.022 (1.013 to 1.031)	1.019 (1.010 to 1.028)	1.017 (1.008 to 1.026)
Mean duration of LIPA bouts (min)	0.753 (0.582 to 0.974)	0.776 (0.601 to 1.002)	0.797 (0.618 to 1.028)
Mean duration of MVPA bouts (min)	0.786 (0.685 to 0.901)	0.814 (0.712 to 0.932)	0.848 (0.742 to 0.969)
Intensity constant	1.491 (1.279 to 1.739)	1.434 (1.230 to 1.672)	1.38 (1.177 to 1.618)
Intensity gradient	0.28 (0.184 to 0.426)	0.316 (0.207 to 0.482)	0.353 (0.227 to 0.550)
Timing of the most active 5h window (h)	0.989 (0.930 to 1.052)	0.982 (0.924 to 1.045)	0.981 (0.922 to 1.044)

*Models adjusted for age (timescale), sex, ethnicity, marital status, education, and last occupational position.

†Models additionally adjusted for smoking status, alcohol consumption, and fruit and vegetable consumption.

‡Models additionally adjusted for body mass index, hypertension, hyperlipidaemia, diabetes, and morbidity index.

Abbreviations: LIPA, light intensity physical activity; MVPA, moderate-to-vigorous activity; SB, sedentary behaviour.

Yerramalla MS, *et al. BMJ Open Sp Ex Med* 2024; 10:e001873. doi: 10.1136/bmjsem-2023-001873

eTable 3 Correlation matrix of 13 features of daily movement behaviours

	[1]	[2]	[3]	[4]	[5]	[6]	[7]	[8]	[9]	[10]	[11]	[12]	[13]
[1] Average acceleration	1.00												
[2] Total duration of SB	-0.81	1.00											
[3] Total duration of LIPA	0.71	-0.78	1.00										
[4] Total duration of MVPA	0.89	-0.66	0.48	1.00									
[5] Number of sedentary bouts	-0.58	0.63	-0.71	-0.40	1.00								
[6] Number of LIPA bouts	0.35	-0.54	0.72	0.12	-0.37	1.00							
[7] Number of MVPA bouts	0.40	-0.17	-0.02	0.54	-0.12	-0.10	1.00						
[8] Mean duration of sedentary bouts	0.51	-0.51	0.78	0.27	-0.78	0.28	-0.03	1.00					
[9] Mean duration of LIPA bouts	0.75	-0.72	0.90	0.58	-0.79	0.38	0.05	0.91	1.00				
[10] Mean duration of MVPA bouts	0.84	-0.73	0.65	0.89	-0.49	0.25	0.20	0.41	0.73	1.00			
[11] Intensity constant	0.75	-0.59	0.41	0.77	-0.50	0.18	0.62	0.29	0.49	0.65	1.00		
[12] Intensity gradient	-0.64	0.48	-0.22	-0.67	0.34	-0.06	-0.65	-0.13	-0.29	-0.50	-0.97	1.00	
[13] Timing of the most active 5h window	0.05	-0.04	0.02	0.04	-0.01	-0.04	0.02	0.05	0.04	0.03	0.03	-0.04	1.00

Abbreviations: LIPA, light intensity physical activity; MVPA, moderate-to-vigorous physical activity; SB, sedentary behaviour.

Yerramalla MS, *et al. BMJ Open Sp Ex Med* 2024; 10:e001873. doi: 10.1136/bmjsem-2023-001873

eTable 4 Parameters assessing the variability of participants within and between clusters for different profiles

Number of profiles	Total within cluster sum of squares*	Between clusters sum of squares†
**3**	24909.46	27181.54
**4**	28539.43	23551.57
**5**	22564.41	29526.59

* The distance between participants within the same profile, with a lower value (favourable) indicating that participants are similar within the assigned profile.

†The distance between cluster, with a higher value (favourable) indicating that participants from different profiles are dissimilar.

Yerramalla MS, *et al. BMJ Open Sp Ex Med* 2024; 10:e001873. doi: 10.1136/bmjsem-2023-001873

eTable 5 Standardized values of movement behaviour variables by profiles

Daily movement behaviour features	Active (N = 726)	Active sitters (N = 890)	Light movers (N = 1033)	Prolonged sitters (N = 1040)	Most sedentary (N = 302)
Average acceleration (m*g*)	1.352 (0.785)	0.353 (0.780)	-0.008 (0.318)	-0.800 (0.272)	-1.513 (0.293)
Total duration of SB (min/day)	1.320 (0.607)	0.062 (0.619)	0.211 (0.540)	-0.729 (0.562)	-1.568 (0.691)
Total duration of LIPA (min/day)	1.259 (0.714)	-0.297 (0.478)	0.555 (0.513)	-0.698 (0.445)	-1.645 (0.478)
Total duration of MVPA (min/day)	1.232 (0.930)	0.614 (0.755)	-0.309 (0.429)	-0.718 (0.34)	-1.242 (0.170)
Number of sedentary bouts	0.778 (0.760)	-0.239 (0.600)	0.682 (0.705)	-0.523 (0.636)	-1.695 (0.807)
Number of LIPA bouts	1.232 (0.582)	-0.131 (0.509)	0.493 (0.502)	-0.706 (0.48)	-1.831 (0.619)
Number of MVPA bouts	1.389 (0.854)	0.248 (0.667)	-0.060 (0.542)	-0.721 (0.405)	-1.381 (0.232)
Mean duration of sedentary bouts (min)	0.709 (0.212)	0.052 (0.374)	0.441 (0.233)	-0.343 (0.48)	-2.186 (2.159)
Mean duration of LIPA bouts (min)	0.650 (0.990)	-0.355 (0.741)	0.474 (0.876)	-0.336 (0.816)	-0.978 (0.911)
Mean duration of MVPA bouts (min)	0.189 (0.657)	0.957 (1.244)	-0.393 (0.425)	-0.288 (0.668)	-0.937 (0.998)
Intensity constant	0.794 (0.545)	0.918 (0.602)	-0.225 (0.461)	-0.566 (0.573)	-1.894 (0.845)
Intensity gradient	0.585 (0.675)	1.010 (0.778)	-0.369 (0.512)	-0.466 (0.649)	-1.517 (0.806)
Timing of the most active 5h window (h)	0.082 (1.046)	0.069 (1.032)	-0.030 (0.939)	-0.090 (0.941)	0.013 (1.152)

Data are mean and standard deviation of standardized values. Mean value of 0 corresponds to the average observed value in the study population. Positive values represent higher acceleration, higher total duration in SB, LIPA and MVPA, higher number of bouts, higher mean duration of bouts, higher intensity gradient, higher intensity constant, and later timing of activity.

Abbreviations: LIPA, light intensity physical activity; MVPA, moderate-to-vigorous activity; SB, sedentary behaviour.

Yerramalla MS, *et al. BMJ Open Sp Ex Med* 2024; 10:e001873. doi: 10.1136/bmjsem-2023-001873

eTable 6 Association between profiles of movement behaviours and all-cause mortality, with different reference categories (N total = 3991, N cases = 410, mean follow-up (SD) = 8.1 (1.3) years)

	**Hazard ratio (95% confidence interval) ***			
**Profiles**	**Reference: Active sitters**	**Reference: Light movers**	**Reference: Prolonged sitters**	**Reference: Most sedentary**
Active	0.64 (0.41 to 0.99)	0.57 (0.38 to 0.86)	0.60 (0.40 to 0.90)	0.31 (0.20 to 0.48)
Active sitters	1.00 [Reference]	0.90 (0.64 to 1.25)	0.94 (0.68 to 1.31)	0.48 (0.34 to 0.70)
Light movers	1.12 (0.80 to 1.56)	1.00 [Reference]	1.05 (0.81 to 1.37)	0.54 (0.40 to 0.73)
Prolonged sitters	1.06 (0.77 to 1.48)	0.95 (0.73 to 1.24)	1.00 [Reference]	0.51 (0.39 to 0.68)
Most sedentary	2.07 (1.44 to 2.97)	1.85 (1.37 to 2.50)	1.95 (1.47 to 2.57)	1.00 [Reference]

*All models adjusted for age (time-scale), sex, ethnicity, marital status, education, last occupational position, smoking status, alcohol consumption, fruit and vegetable consumption, body mass index, hypertension, hyperlipidaemia, diabetes, and morbidity index.

Yerramalla MS, *et al. BMJ Open Sp Ex Med* 2024; 10:e001873. doi: 10.1136/bmjsem-2023-001873

eTable 7 Association between profiles of movement behaviours and all-cause mortality using 2-year washout period (N total = 3946, N cases = 365, mean follow-up (SD) = 6.2 (1.04) years)

		Hazard ratio (95% confidence interval)		
**Profiles**	**N cases/N total**	**Model adjusted for sociodemographic factors***	**Additionally adjusted for lifestyle factors†**	**Additionally adjusted for health-related factors‡**
Active	29/724	1.00 [Reference]	1.00 [Reference]	1.00 [Reference]
Active sitters	50/883	1.46 (0.92 to 2.30)	1.46 (0.92 to 2.31)	1.47 (0.93 to 2.33)
Light movers	93/1021	1.71 (1.12 to 2.60)	1.70 (1.12 to 2.59)	1.69 (1.11 to 2.58)
Prolonged sitters	112/1026	1.72 (1.13 to 2.60)	1.66 (1.10 to 2.52)	1.62 (1.06 to 2.47)
Most sedentary	81/292	3.60 (2.32 to 5.58)	3.49 (2.24 to 5.42)	3.25 (2.06 to 5.13)

*Models adjusted for age (time-scale), sex, ethnicity, marital status, education, and last occupational position.

†Models additionally adjusted for smoking status, alcohol consumption, and fruit and vegetable consumption.

‡Models additionally adjusted for body mass index, hypertension, hyperlipidaemia, diabetes, and morbidity index.

.

Yerramalla MS, *et al. BMJ Open Sp Ex Med* 2024; 10:e001873. doi: 10.1136/bmjsem-2023-001873

SUPPLEMENTARY FIGURES

eFigure 1 Participant flow chart

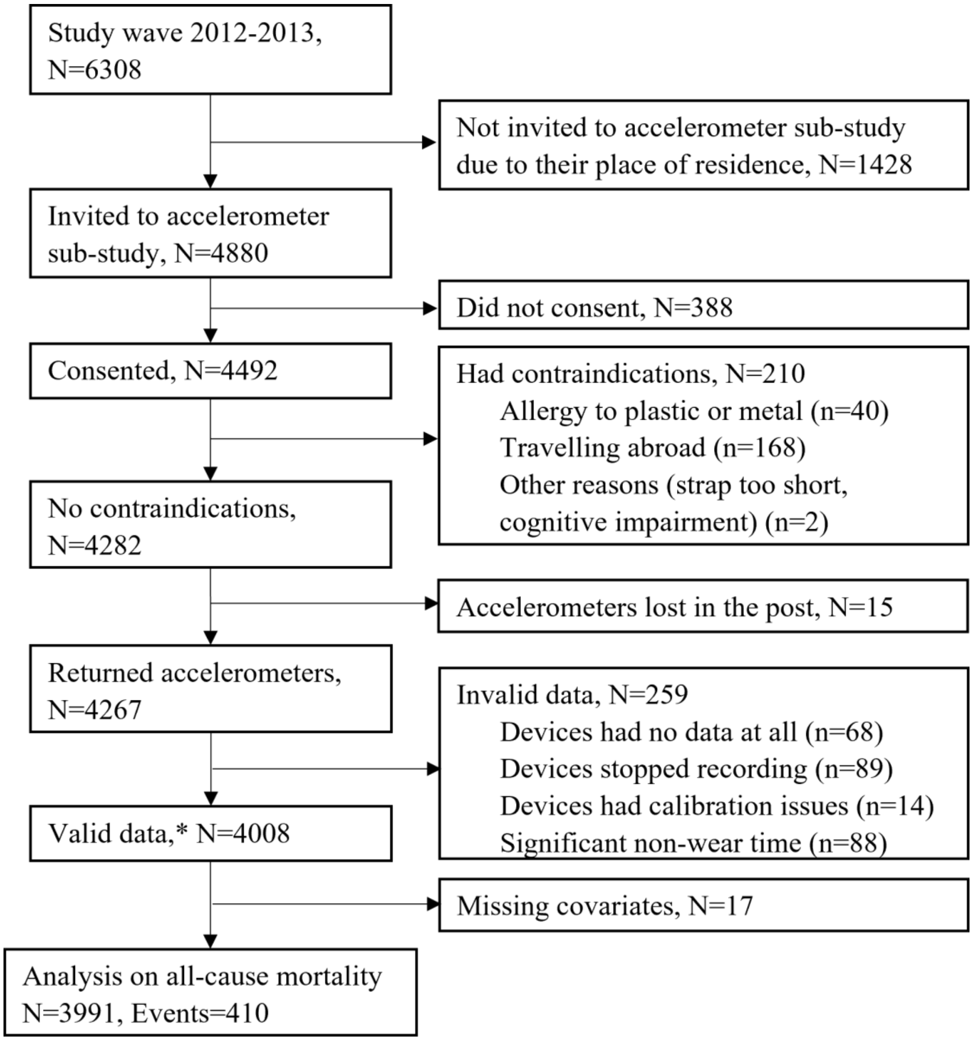

*Defined as accelerometer wear time ≥2/3 of waking period, for at least 2 weekdays and 2 weekend days.

Yerramalla MS, *et al. BMJ Open Sp Ex Med* 2024; 10:e001873. doi: 10.1136/bmjsem-2023-001873

eFigure 2 Determination of the optimal number of clusters

A) Using the Elbow method

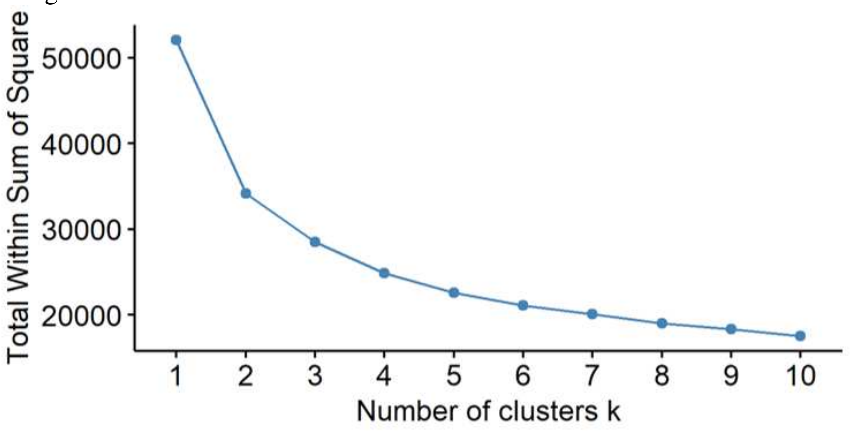

B) Using the Gap statistic method

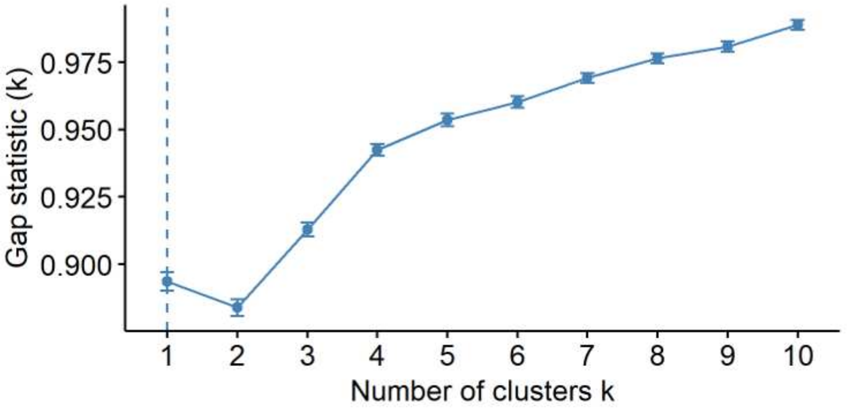

A) The Elbow method selects the number of clusters to be such that adding an additional cluster does not significantly reduce the within-group sum of squares, which quantifies the degree to which items within a cluster are similar, representing a trade-off between a reasonable number of clusters and clustering quality. B) Gap statistic method compares the clusters created from the observed data and clusters created from a randomly generated dataset, known as the reference dataset. For a given k, the gap statistic is the difference in the total within-cluster variance for the observed data and that of the reference dataset. The optimal number of clusters is denoted by the value of k that yields the largest gap statistic.

Yerramalla MS, *et al. BMJ Open Sp Ex Med* 2024; 10:e001873. doi: 10.1136/bmjsem-2023-001873

eFigure 3 Visualization of the five profiles of daily movement behaviours using the initial two principal components of the principal component analysis

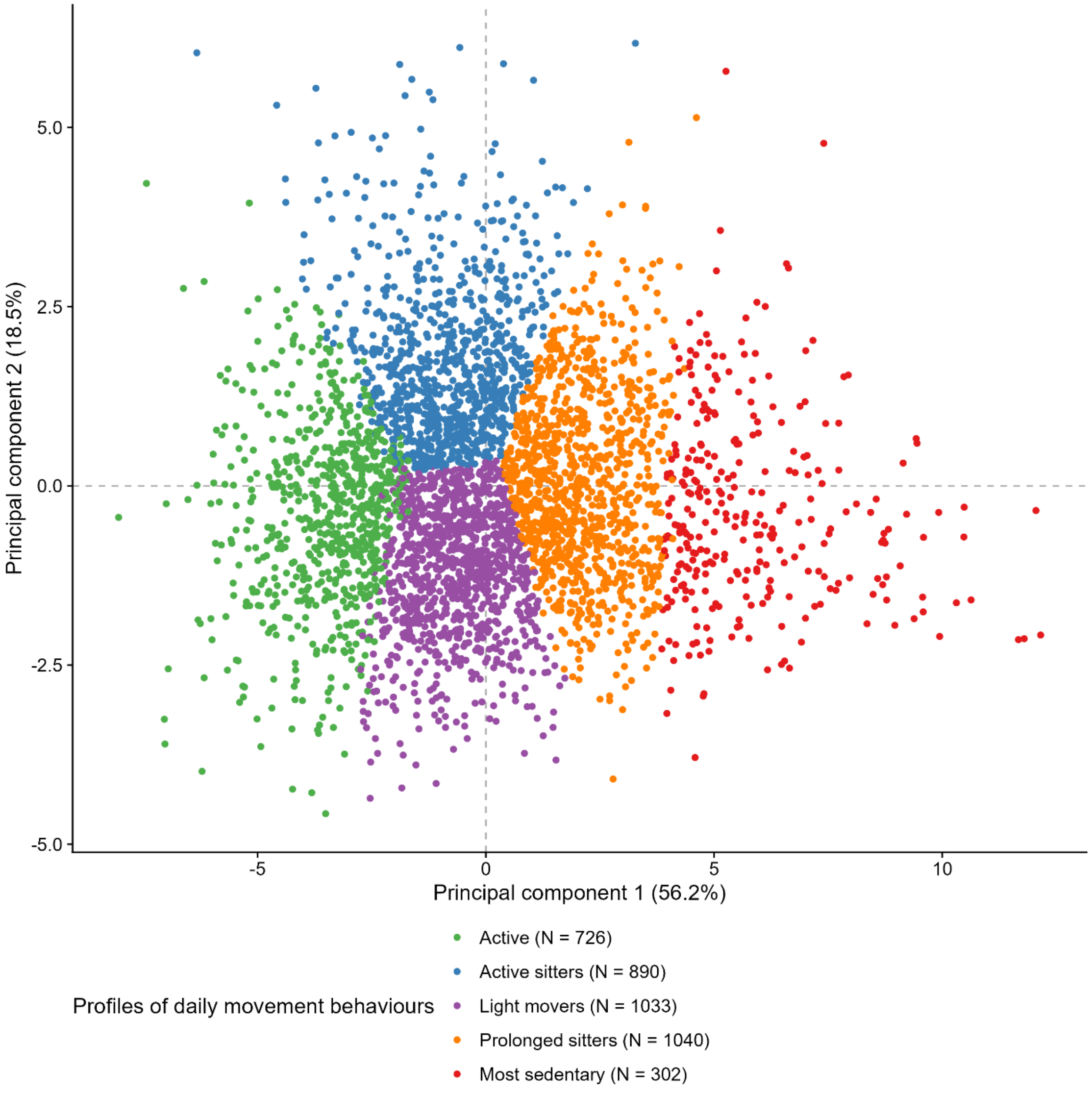

Note: The values in parentheses correspond to the proportion of variance explained in the dataset by each principal component.

Yerramalla MS, *et al. BMJ Open Sp Ex Med* 2024; 10:e001873. doi: 10.1136/bmjsem-2023-001873

eFigure 4 Loadings of movement behaviour features in the two first principal components used to describe the identified movement behaviour clusters in eFigure 3.

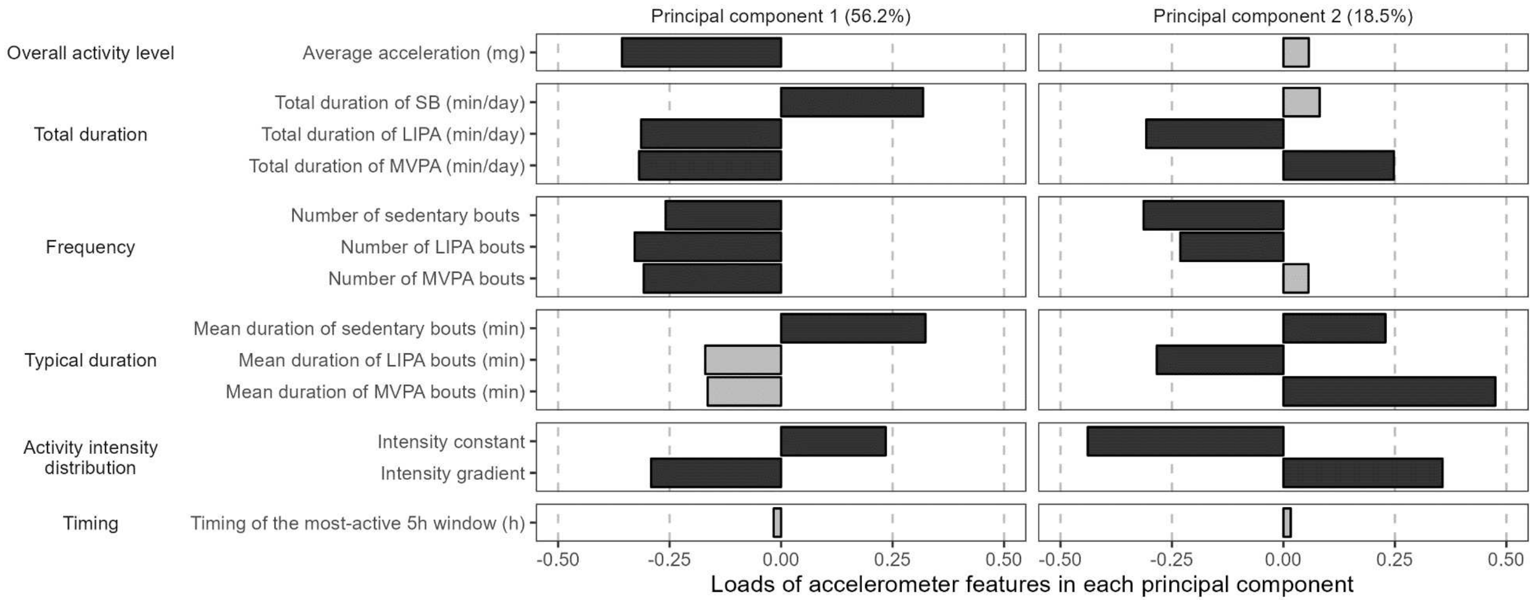

Note: Features with loading absolute values above 20 (in black grey on the graph) are considered to contribute considerably to the principal. The values in parentheses correspond to the proportion of variance explained in the dataset by each principal component.

Higher values for principal component 1 correspond to lower average acceleration, more time in SB and less in LIPA and MVPA, less number of bouts of (in)activity, less fragmented sedentary time (as denoted by longer mean duration of sedentary bouts), higher intensity constant, and lower gradient denoting more time in inactivity and time accumulated during waking period is less evenly distributed across the intensity spectrum.

Higher values for principal component 2 correspond to less time in LIPA and more in MVPA, less number of sedentary and LIPA bouts, more fragmented sedentary increased number of sedentary and MVPA bouts and lower number of LIPA bouts, lower time in inactivity, and time more evenly distributed across the intensity spectrum.

Abbreviations: PA: physical activity; SB: sedentary behaviour; MVPA: moderate-to-vigorous physical activity; LIPA: light-intensity physical activity.

Yerramalla MS, *et al. BMJ Open Sp Ex Med* 2024; 10:e001873. doi: 10.1136/bmjsem-2023-001873
